# Myxedema psychosis: A protocol for a systematic review and a pooled analysis

**DOI:** 10.1097/MD.0000000000020778

**Published:** 2020-06-26

**Authors:** Mouhand F.H. Mohamed, Timo Siepmann, Salah Suwileh, Mohammed H. Mohammed, Samreen Mohamed, Lina O. Abdalla, Abdel-Naser Elzouki, Mohamed H. Mahmoud, Dabia Al-Mohanadi, Mohammed Danjuma

**Affiliations:** a Internal Medicine Department, Hamad Medical Corporation, Doha, Qatar; b Dresden International University Division of Health Care Sciences Center for Clinical Research and Management Education Dresden; c Department of Neurology, University Hospital Carl Gustav Carus, Technische Universität Dresden, Dresden, Germany; d Psychiatry Department, Hamad Medical Corporation; e College of Medicine, Qatar University; f Primary Health Care Centers; g Endocrine Department, Hamad Medical Corporation, Doha, Qatar.

**Keywords:** hypothyroidism, madness, myxedema, psychosis, systematic review

## Abstract

**Background:**

Myxedema psychosis (MP) is a rare presentation of hypothyroidism. Although known for >70 years, a significant lack of systematic literature describing this condition exists. This limits the clinician's ability to identify and manage this entity properly. Hence, we aimed to systematically review the literature and summarize the presentation, diagnosis, management, and outcomes of this rare entity.

**Methods:**

Systematic review following PRISMA guidance. We will perform a comprehensive search of PubMed, Medline, Embase, Google Scholar (first 300 hits), and Cochrane databases for published observational studies, case series, and case reports. We will use descriptive statistics to provide summary estimates of demographics, common presenting features, laboratory test results, imaging findings, treatment administered, and outcomes. Moreover, continuous variables will be compared by the Wilcoxon Mann Whitney test, whereas categorical variables will be assessed by the *χ*^2^ test. Bivariate and multivariate regression will be performed to assess risk factors associated with poor outcome. A scoping review revealed that a meta-analysis might not be feasible owing to the paucity of systematic studies describing the condition.

**Results:**

This is the first systematic review examining this rare entity. Thus, the result of which will be significant. We hope that this review will help in identifying relevant predictive clinical or laboratory characteristics. Additionally, it identifies the best treatment strategies. The findings of this review will help increase our knowledge of this condition so as to recognize this condition promptly. Also, it will assist in differentiating MP from masqueraders, such as Hashimoto encephalopathy (HE). The results of this review will be published in a peer-reviewed journal.

**Conclusion:**

This is the first systematic review exploring MP demographics, diagnosis treatment, and outcomes. The information gathered by this review will be necessary for patients, clinicians, researchers, and guideline makers.

**PROSPERO registration number:**

CRD42020160310

## 1 Introduction

Hypothyroidism is a prevalent condition affecting up to 3.6% of the world population.^[1]^ In 1949 Asher et al^[2]^ linked the development of psychosis and hypothyroidism in his description of 14 cases. Myxedema madness was the initial term described by Asher; later, myxedema psychosis (MP) emerged as a better description of this entity. MP is considered a form of secondary psychosis.^[3]^ Today more than half a century since its first description, little is known about the diagnosis and management of this entity.^[4]^

MP is not the only cause of neuropsychiatric changes in patients with hypothyroidism. Hashimoto encephalopathy (HE) is an autoimmune disease that can also lead to psychosis in this cohort of patients, although it has certain specific features, clinicians may mistakenly confuse it with MP. The 2 conditions vary in their presentations, pathogenesis, and, more importantly, in their management strategy.^[5]^ Guided by the aforementioned discussion, we planned to systematically review the relevant literature describing MP demographics, diagnosis, management strategies, and the outcomes. Our objective was to answer this set of questions:

1. What are the demographic characteristics and clinical features of MP?
2. What are the expected laboratory tests and imaging findings?
3. What constitutes optimal management strategies in patients with MP? Moreover, whether there was demonstrable superiority of one approach over the other?
4. What are the expected outcomes following adequate therapy? Furthermore, are these dependent on any discernible predictor variable (s)?

## 2 Methods

This protocol follows PRISMA-P guidance.^[6]^ The review is registered in the International Prospective Register of Systematic Reviews (registration number: CRD42020160310).

## 3 Eligibility criteria

### 3.1 Type of studies

We will include case studies, case series, and observational studies describing MP. Moreover, we will exclude studies published before 1980, studies not written in the English language, and studies reporting on the pediatric population.

### 3.2 Type of participants

Adults >18 years were diagnosed with MP evident by psychotic features. Clinical hypothyroidism should be confirmed by an abnormally elevated TSH, in addition, the presence of clinical hypothyroidism's features or low thyroid hormones. We will exclude HE, thyroxine-induced mania, secondary hypothyroidism, and subclinical hypothyroidism.

### 3.3 Type of Intervention

Hypothyroidism resulting in psychosis is our exposure of interest. Oral thyroxine (T4) whenever feasible will be examined versus other thyroid hormone replacement formulations and route as interventions.

### 3.4 Type of comparator

Intravenous (IV) T4 or triiodothyronine (T3).

### 3.5 Type of outcome measures

#### 3.5.1 Primary outcome

We will summarize various demographics, clinical features, laboratory, and imaging findings—additionally, treatments administered and outcomes.

#### 3.5.2 Secondary outcomes

Recovery rate as a categorical outcome. Delayed recovery (>2 weeks) as a categorical outcome. Also, the median days to recovery as a continuous variable.

### 3.6 Information source and literature search

We will search the following databases: PubMed, Medline, EMBASE, Google Scholar (first 300 hits), and Cochrane databases for studies published. We will limit our search to articles written in the English language only. We will use free text, keywords, emtree, and MesH terms. Example of a proposed PubMed search strategy: ((psychosis[MeSH Terms]) OR psychosis[Title/Abstract]) OR psycho∗[Title/Abstract]) OR madness[Title/Abstract]) OR psychiatric[Title/Abstract]) AND English[lang])) AND ((((((hypothyroidism[MeSH Terms]) OR hypothyroid∗[Title/Abstract]) OR myxedema[MeSH Terms]) OR myxedema∗[Title/Abstract]) OR myxoedema∗[Title/Abstract]) AND English[lang]). Additionally, a relevant citation and references will be conducted.

### 3.7 Screening and data extraction

Two reviewers (MFHM) and (SS) will perform the screening in 2 steps. First, the titles and abstracts of retrieved articles will be screened for eligibility. Potentially relevant articles will then be retrieved for full-text review and screening. Discrepancies in article selection will be solved by a discussion between authors. Disagreement not settled by discussion will be settled by a third reviewer (MID). We will use excel proforma to collect relevant data such as article type, author, year, patients’ sex, age, sex, presenting features, the type of psychiatric presentation, treatment administered, and outcomes and so on.

### 3.8 Risk of bias assessment

We will use a novel tool for assessing the quality of case reports.^[7]^ New Castle Ottawa tool will be used to assess the quality of observational studies.^[8]^ If a meta-analysis was populated, then funnel plots will be generated to ascertain publication bias. GRADE system will be used to assess the strength of evidence of the meta-analytical results.

### 3.9 Statistical analysis

Anticipating finding case studies only, we will perform a pooled analysis. We will use descriptive statistics to provide summary estimates using the median (interquartile range) for continuous variables and frequencies for categorical variables. Planned comparison between categorical variables using the *χ*^2^ test and Mann–Whitney for continuous data will be attempted. Additionally, predictors of outcome will be ascertained utilizing bi- and multivariate logistic regression. Our scoping review, as explained earlier, did not reveal the presence of systematic studies. However, if found, then we will attempt to perform a quantitative synthesis utilizing the random-effects model. *I*^2^ will be used then to ascertain heterogeneity of which heterogeneity of >60% will be considered marked. Odds ratios will be used as a pooling measure of effect in the case of meta-analysis. We will use Jamovi 1.1.9 for statistical analysis.^[9]^

### 3.10 Subgroup analysis and sensitivity analysis

If the number of studies permits, we will perform a subgroup analysis based on the type and route of thyroid hormone supplements administered.

### 3.11 Ethics and Dissemination

Ethical approval is not required for the purposes of our review, as it is a secondary analysis of already available data. The results of our review will be disseminated in conferences and published in a peer-reviewed journal.

## 4 Discussion

This review will be the first systematic attempt to study MP. It will provide the medical community with a holistic overview of this condition. The findings from our review will be relevant to clinicians taking care of patients suspected or confirmed to have this entity. Moreover, it will be of extreme value to researchers interested in studying this disease and guideline makers. Additionally, it will be of value in devising standardized diagnostic criteria for both MP and its mimicker HE. Foreseen limitations of our review are the absence of sizeable observational studies or systematic, controlled studies, varying definitions of psychosis, underreporting in case studies. Furthermore, any attempted analysis will depend on finding a good number of cases; otherwise, such analysis may be underpowered to detect differences in outcome occurrence between groups. Nonetheless, our review will constitute the best available evidence until further data accrue.

## Acknowledgment

The authors acknowledge the Qatar National Library (QNL) for funding the open access fees of this publication.

## Author contributions

**Conceptualization:** Mouhand F.H Mohamed

**Data curation:** Mouhand F.H Mohamed, Salah Swuileh, Mohammed H. Mohammed, Samreen Mohamed, Lina O. Abdalla

**Formal analysis:** Mouhand F.H Mohamed, Mohammed Danjuma

**Methodology** Mouhand F.H Mohamed, Mohammed Danjuma

**Project administration:** Mouhand F.H Mohamed, Salah Swuileh,

**Resources** Abdel-Naser Elzouki, Dabia Al-Mohanadi

**Review & editing:** Mouhand F.H Mohamed, Mohammed Danjuma, Abdel-Naser Elzouki, Dabia Al-Mohanadi, Mohamed H. Mahmoud

**Supervision:** Timo Siepmann

**Writing – original draft** Mouhand F.H Mohamed
